# Dr. S. W. Butler

**Published:** 1874-01

**Authors:** 


It is with painful surprise that we learn just as we are going to press, of the death of Dr. S. W. BUTLER, editor of *The Philadelphia Medical and Surgical Reporter.* Dr. BUTLER has been the editor of this Journal ever since its first publication in 1858, and was previously connected as Assistant Editor and Editor with the *New Jersey Medical Reporter.*

His long Editorial life has made him generally known to the profession of the United States, and his death will be mourned by many friends. Dr. Butler at the time of his death was over fifty years of age. The disease of which he died was Phthisis Pulmonalis.

